# Supplementary material for: Bioactive Constituents of Verbena officinalis Alleviate Inflammation and Enhance Killing Efficiency of Natural Killer Cells
Source: Int J Mol Sci. 2023 Apr 12;24(8):7144. doi: 10.3390/ijms24087144 (PMC10138337; doi:10.3390/ijms24087144)
Supplement: Supplementary file 1 [file ijms-24-07144-s001.zip › ijms-2215600-supplementary.pdf]

Supplementary Information

Scheme S1. Chemical constituents of VO extract detected by UPLC-Q-TOF-MS.

No.	Identification	Elemental composition	RT	Calculated mass	m/z	Fragment ions	ppm
1	3,4-dihydroverbenalin	$C_{17}H_{26}O_{10}$	10.065	390.1526	413.1480[M+Na]$^+$	105.0716, 179.0733	-14.96
2	Verbeofflin I	$C_{11}H_{12}O_6$	10.210	240.0634	239.0559[M-H]$^-$	139.0386, 147.0462, 195.0650	0.89
3	Hastatoside	$C_{17}H_{24}O_{11}$	10.214	404.1319	403.1219[M-H]$^-$	195.0650, 223.0600, 241.0713	-1.21
					427.1216[M+Na]$^+$	165.0570, 343.0921	
4	Verbenalin	$C_{17}H_{24}O_{10}$	10.794	388.1369	387.1312[M-H]$^-$	101.0242, 179.0471, 225.0761	-1.29
					411.1267[M+Na]$^+$	149.0621, 167.0728, 195.0681	
5	Quercetin	$C_{15}H_{10}O_7$	13.112	302.0427	301.0341[M-H]$^-$	151.0051, 161.0271, 179.0370	4.24
					303.0554[M+H]$^+$	163.0410, 195.0689	
6	Acteoside	$C_{29}H_{36}O_{15}$	14.522	624.2054	623.1991[M-H]$^-$	161.0249, 179.0338, 461.1627, 487.1425	-1.53
					647.2083[M+Na]$^+$	163.0420, 325.0980, 471.1586	
7	Luteolin	$C_{15}H_{10}O_6$	14.588	286.0477	285.0395[M-H]$^-$	255.0278, 283.0240	3.37
					287.0607[M+H]$^+$	139.0051, 153.0198	
8	Isorhamnetin	$C_{16}H_{12}O_7$	14.986	316.0583	315.0493[M-H]$^-$	283.0230, 300.0252	5.48
9	Luteolin 7-*O*-*β*-gentiobioside	$C_{27}H_{30}O_{16}$	15.169	610.1534	609.1416[M-H]$^-$	179.0375, 254.9867, 283.0317	7.4
10	Isoacteoside	$C_{29}H_{36}O_{15}$	15.898	624.2054	623.1970[M-H]$^-$	161.0245, 179.0333, 461.1645	1.84
					647.2072[M+Na]$^+$	163.0417, 325.0976	
11	Leucosceptoside A	$C_{30}H_{38}O_{15}$	16.114	638.2211	637.2177[M-H]$^-$	161.0265, 175.0410, 461.1702	-6.13
					661.2244[M+Na]$^+$	163.0421, 177.0552, 322.1341	
12	Apigenin	$C_{15}H_{10}O_5$	16.296	270.0528	269.0449[M-H]$^-$	161.0235, 179.0337	2.4
					271.0651[M+H]$^+$	163.0414	
13	Kaempferol	$C_{15}H_{10}O_6$	16.960	286.0477	285.0392[M-H]$^-$	161.0248, 179.0337	4.43

Supplementary Movie S1. Verbenalin treatment reduces time required for killing and enhances average kills per NK. Primary human NK cells were stimulated with IL-2 in the presence of Verbenalin with indicated concentrations for 3 days prior to experiments. Target cells (K562-pCasper) were embedded in collagen and NK cells were added from the top. Killing events were visualized at 37 °C every 70 sec. One representative NK cell from each condition (0 µM vs. 30 µM) is shown. NK cells were not fluorescently labeled and marked with blue tracks. The target cells in contact with the corresponding NK cell are numbered.

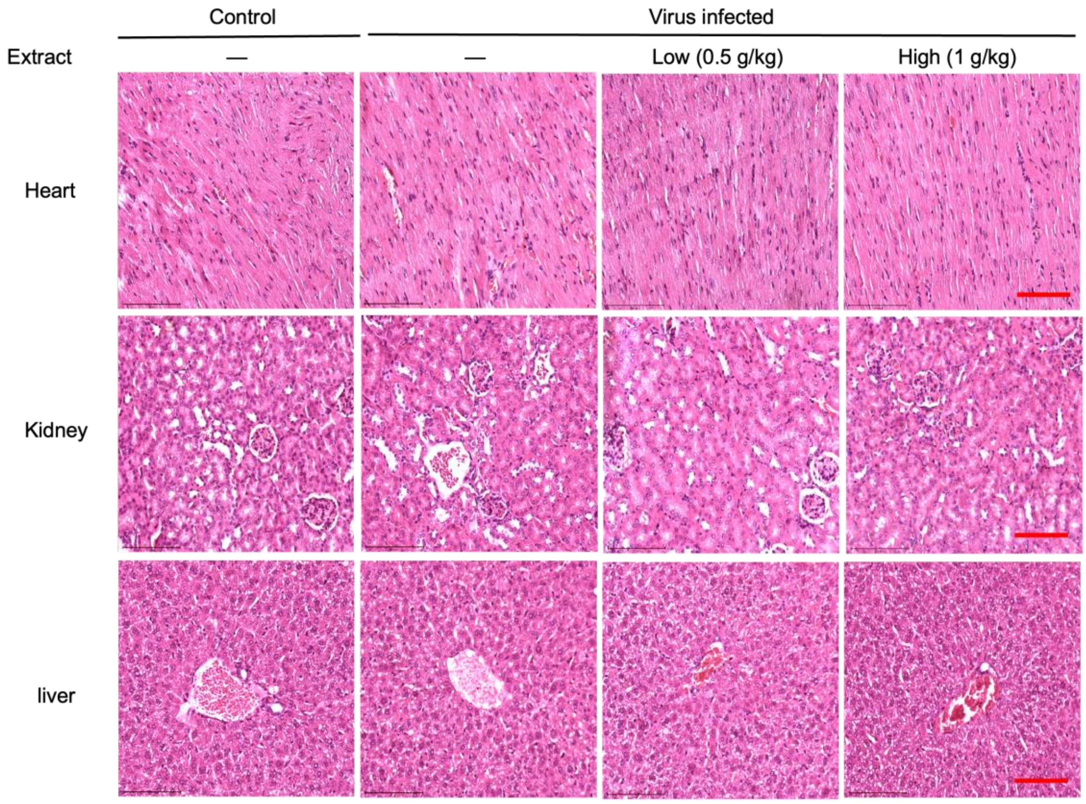

Supplementary Figure S1. Assessment of the potential toxicity of the low and high doses of VO extract on the heart, kidney, and liver. C57BL/6J mice were intranasally challenged with the influenza virus A/PR8/34 (H1N1) (100 PFU) on day 0 and subsequently orally administered a single dose of VO extract (low dose/Low: 0.5 g/kg; high dose/High: 1 g/kg) every day for 3 days. On day 3, the mice were euthanized, and their heart, kidney, and liver tissues were collected, fixed in 10% buffered formalin, and embedded in paraffin. Each tissue was cut into 4 µm sections and stained with hematoxylin and eosin. The sections were analyzed using an optical microscope with a 200× magnification, with *n* = 6 for each group. Scale bars are 100 µm.

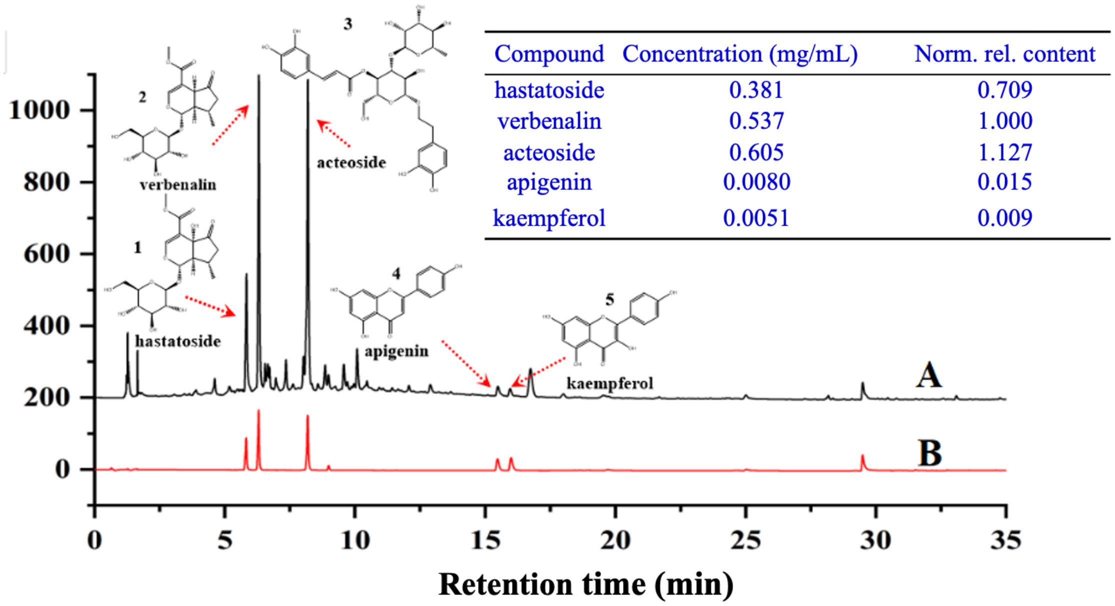

Compound	Concentration (mg/mL)	Norm. rel. content
hastatoside	0.381	0.709
verbenalin	0.537	1.000
acteoside	0.605	1.127
apigenin	0.0080	0.015
kaempferol	0.0051	0.009

Supplementary Figure S2. Determination of relative content of key bioactive components in *Verbena officinalis* (VO) extract. UPLC chromatograms were used to determine the relative content of Hastatoside, Verbenalin, Acteoside, Apigenin, and Kaempferol in the extract. Sample volumes of 2 µL were used and UV detection was performed at 254 nm. Representative curves for the VO extract (10 mg/mL) and for a mixture of standard substances are shown in panels A and B, respectively. The mixture of standard substances contains the following purified substances: Hastatoside (0.1 mg/mL), Verbenalin (0.1 mg/mL), Acteoside (0.1 mg/mL), Apigenin (0.01 mg/mL), and Kaempferol (0.1 mg/mL). Both the VO extract and standard substances were dissolved in methanol. The concentrations and normalized relative content to Verbenalin (Norm. rel. content) are shown in the table.

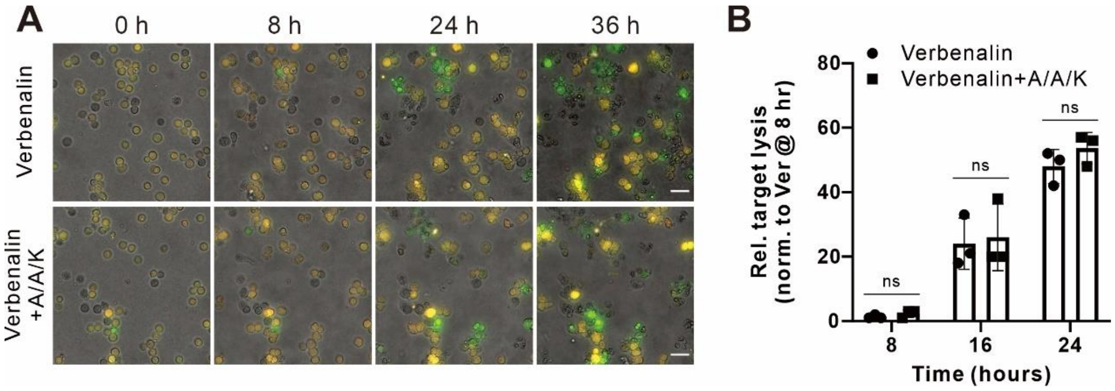

Supplementary Figure S3. The presence of the three negative bioactive constituents does not counteract the effect of Verbenalin on NK cell killing. Primary human NK cells were cultured with Verbenalin alone (30 µM) or Verbenalin (30µM) with the three negative bioactive components in VO extract (A/A/K: Acteoside, Apigenin, and Kaempferol) for three days in the presence of IL-2 (100 U/mL). The concentrations of Acteoside, Apigenin, and Kaempferol were 1.127, 0.015, and 0.009 times that of Verbenalin, respectively. The killing kinetics were determined using 3D real-time killing assay. K562-pCasper target cells were embedded in collagen matrices, and NK cells were added from the top. The killing events were visualized at 37 °C every 20 min for 24 h. Yellow indicates live target cells. Turning green indicates that they were undergoing apoptosis. Fully lysed target cells lost fluorescence signals. The time lapse from one representative donor is shown in **A** with scale bars of 40 µm. The target lysis at the indicated time points was normalized to the Verbenalin-treated group at 8 h and is shown in **B**. Results are from three donors.

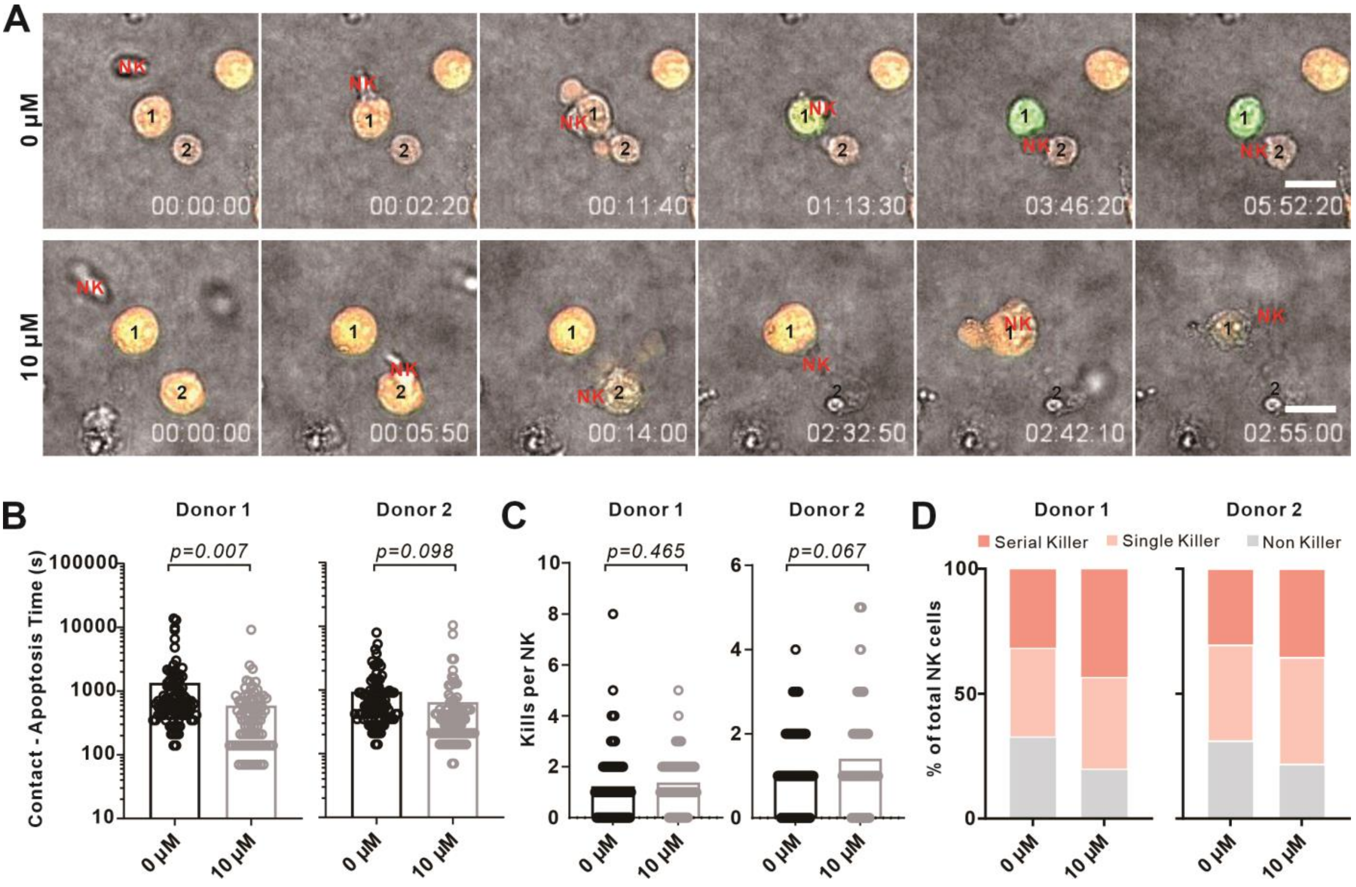

Supplementary Figure S4. Verbenalin accelerates NK cell killing and increases killing events per NK cell. Primary human NK cells were stimulated with IL-2 in the presence of Verbenalin (10 µM) for 3 days prior to experiments. K562-pCasper target cells were embedded in collagen and NK cells were added from the top. Killing events were visualized at 37 °C every 70 sec for 14 h. (A) NK cells make multiple contacts with target cells. One representative NK cell from each condition is shown. NK cells (marked in red) were not fluorescently labeled. The corresponding target cells in contact are numbered. Scale bars are 20 µm. (B) Verbenalin shortens the time required for NK cell killing. The time from NK/target contact to target cell apoptosis was quantified. (C) Verbenalin treatment enhances the number of target cells killed per NK cell. Both apoptosis and necrosis were considered as killing events. (D) The fraction of serial killers is elevated by Verbenalin treatment. The fraction of serial killers (NK cells that killed more than one target cell), single killers (NK cells that killed only one target cell), and non-killers (NK cells that did not kill any target cells) for each donor was analyzed. Results are from two donors. Around 70 NK cells were randomly chosen from each condition. Statistical analysis was performed using a Mann–Whitney test.

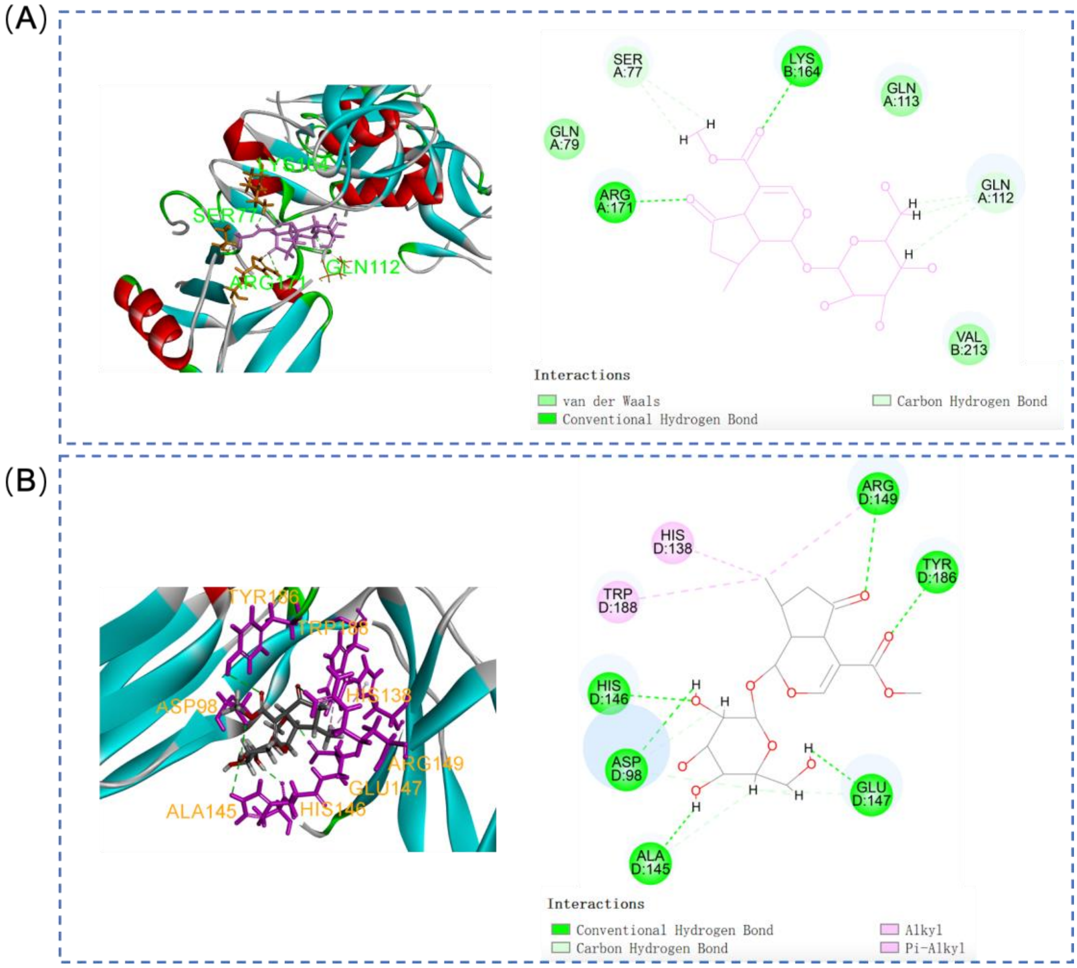

Supplementary Figure S5. Molecular docking of Verbenalin with inhibitory receptors of NK cells. (A) Molecular dynamic docking of Verbenalin with NKG2A (PDB ID: 3BDW; left panel) and intermolecular interactions between Verbenalin and NKG2A (right panel). (B) Molecular dynamic docking of Verbenalin with KIR2DL1 (PDB ID: 1IM9; left panel) and intermolecular interactions between Verbenalin and KIR2DL1 (right panel).
